# The Effect of Iron Ion on the Specificity of Photodynamic Therapy with 5-Aminolevulinic Acid

**DOI:** 10.1371/journal.pone.0122351

**Published:** 2015-03-30

**Authors:** Maiko Hayashi, Hideo Fukuhara, Keiji Inoue, Taro Shuin, Yuichiro Hagiya, Motowo Nakajima, Tohru Tanaka, Shun-ichiro Ogura

**Affiliations:** 1 Graduate School of Bioscience and Biotechnology, Tokyo Institute of Technology, 4259-B47, Nagatsuta-cho, Midori-ku, Yokohama, 226-8501, Japan; 2 Department of Urology, Kochi Medical School, Kohasu, Oko-cho, Nankoku, Kochi, 783-8505, Japan; 3 SBI Pharma CO., LTD., Izumi Garden Tower 20F, 1-6-1, Roppongi Minato-ku, Tokyo, 106-6020, Japan; CINVESTAV-IPN, MEXICO

## Abstract

Recently, photodynamic therapy using 5-aminolevulinic acid (ALA-PDT) has been widely used in cancer therapy. ALA administration results in tumor-selective accumulation of the photosensitizer protoporphyrin IX (PpIX) via the heme biosynthetic pathway. Although ALA-PDT has selectivity for tumor cells, PpIX is accumulated into cultured normal cells to a small extent, causing side effects. The mechanism of tumor-selective PpIX accumulation is not well understood. The purpose of the present study was to identify the mechanism of tumor-selective PpIX accumulation after ALA administration. We focused on mitochondrial labile iron ion, which is the substrate for metabolism of PpIX to heme. We investigated differences in iron metabolism between tumor cells and normal cells and found that the amount of mitochondrial labile iron ion in cancer was lower than that in normal cells. This finding could be because of the lower expression of mitoferrins, which are the mitochondrial iron transporters. Accordingly, we added sodium ferrous citrate (SFC) with ALA as a source of iron. As a result, we observed the accumulation of PpIX only in tumor cells, and only these cells showed sensitivity to ALA-PDT. Taken together, these results suggest that the uptake abilities of iron ion into mitochondria play a key role in tumor-selective PpIX accumulation. Using SFC as a source of iron might thus increase the specificity of ALA-PDT effects.

## Introduction

Recently, photodynamic therapy (PDT) has been widely used in cancer therapy. In PDT, porphyrin derivatives are commonly used to generate singlet oxygen (^1^O_2_) and other reactive oxygen species (ROS) via visible light irradiation [1–3]. One of the most effective photosensitizers for PDT is protoporphyrin IX (PpIX) induced by 5-aminolevulinic acid (ALA) [4,5]. ALA is a naturally occurring amino acid that is synthesized in the body and functions as a biological precursor in the heme biosynthetic pathway. ALA is converted into PpIX in mitochondria. The labile iron ion is then inserted into PpIX to form heme, which is no longer photosensitive, by an enzyme called ferrochelatase. When ALA is administered, the accumulation of PpIX is observed, and it is known that the amount of PpIX after ALA treatment in tumor cells is much higher than that in normal cells. This tumor-selective accumulation of PpIX permits cancer therapy using ALA-PDT.

The amount of PpIX accumulation is reported to be dependent on the activity of ferrochelatase [6,7] and also on the expression of transporters responsible for the import of ALA and export of PpIX [8–10]. Iron metabolism associated with heme biosynthesis is also involved. In mitochondrial iron metabolism, mitoferrin 1 and mitoferrin 2 transport iron ion into mitochondria [11]. Mitoferrin 1 and 2 are homologous members of the mitochondrial solute carrier family. Mitoferrin 1 is expressed mainly in erythroid cells, whereas mitoferrin 2 is expressed in various cells [11]. Iron delivery to ferrochelatase may be mediated by frataxin [12]. A previous study showed that intracellular PpIX accumulation levels were decreased in mitoferrin 2-overexpressing cells [13]. In addition, overexpression of frataxin lowered the accumulation of PpIX [14]. Thus, although several factors are involved in the accumulation of PpIX, the preferential mechanisms are unknown.

Recent *in vitro* research revealed that PpIX is accumulated not only in tumor cells but also in normal cells to a small extent, suggesting the possibility of side effects from the use of ALA-PDT in tumor therapy. The purpose of this study was to elucidate the mechanism of tumor cell-selective PpIX accumulation after ALA treatment and to reduce PpIX accumulation in normal cells.

## Materials and Methods

### Biochemicals

ALA hydrochloride and sodium ferrous citrate (SFC) were purchased from Cosmo Oil Co., Ltd. (Tokyo, Japan). Rhodamine B-{(1,10-phenanthrolin-5-yl) aminocarbonyl} benzyl ester (RPA) was purchased from Squarix biotechnology GmbH (Marl, Germany). Pyridoxal isonicotinoyl hydrazone (PIH) was purchased from Santa Cruz Biotechnology, Inc. (Santa Cruz, CA, USA). RPMI-1640, DMEM (low-glucose), DMEM (high-glucose) media and antibiotic-antimycotic solution (ABAM) were obtained from Nacalai Tesque (Kyoto, Japan). EGM-2 BulletKit was purchased from Lonza group Ltd. (Basel, Switzerland). Fetal bovine serum (FBS) was purchased from Invitrogen (Carlsbad, CA, USA). All other chemicals were of analytical grade.

### Specimens

PDD with oral application of ALA was approved by the ethics committees of Kochi Medical School on December 26, 2006 (No. 18–27) [9]. All patients who were candidates for transurethral biopsy of the bladder or TURBT in the Department of Urology of Kochi Medical School Hospital were enrolled in this study, after written informed consent was obtained. All patients were informed about the potential efficacy as well as the adverse effects of ALA-PDD, such as skin photosensitivity, transient elevation of serum aspartate aminotransferase (AST) and alanine aminotransferase (ALT), nausea, and vomiting in conformity with the Common Terminology Criteria for Adverse Events version 4.0 [15].

### Cells and cell cultures

The human breast adenocarcinoma cell line MCF7 (provided by SBI Pharma CO., LTD., Tokyo, Japan), the normal human mammary epithelial cell line MCF10A (provided by SBI Pharma) and the human gastric cancer cell line MKN45 (provided by Dr. Suzuki, Fukushima medical university, Fukushima, Japan) were maintained in RPMI-1640 medium supplemented with 10% (v/v) FBS and 1% (v/v) ABAM. The human hepatoma cancer cell line HepG2 (purchased from Riken Cell Bank, Tsukuba, Japan) was maintained in DMEM (low glucose) medium supplemented with 10% (v/v) FBS and 1% (v/v) ABAM. The human embryonic kidney 293 cell HEK293 (provided by SBI Pharma CO., LTD., Tokyo, Japan) was maintained in DMEM (high glucose) medium supplemented with 10% (v/v) FBS and 1% (v/v) ABAM. The human umbilical endothelial cell HUVEC (provided by SBI Pharma CO., LTD., Tokyo, Japan) was maintained in EGM-2 BulletKit medium.

### HPLC analysis of PpIX

Cells (1.0 × 10^6^ cells) were incubated with 1 mM ALA with or without 0.5 mM SFC under 5% CO_2_ at 37°C in the dark for 4 h. Cells were washed with phosphate-buffered saline (PBS) and then treated with 200 μl of 0.1 M NaOH. Aliquots of the NaOH-treated cell samples were withdrawn and used for protein concentration assay (Quick Start Bradford protein assay, Bio-Rad Laboratories, Inc., CA, USA), whereas the remaining cellular proteins were denatured by addition of three volumes of solvent A {1 M ammonium acetate, 12.5% acetonitrile (v/v)}:solvent B {50 mM ammonium acetate, 80% acetonitrile (v/v)} (1:9 v/v) solution to the NaOH-treated cell samples. The prepared samples were centrifuged at 10,000 ×*g* for 10 min at 4°C and subjected to high-performance liquid chromatography (HPLC) analysis performed as previously described with some modifications [16–18]. In brief, protoporphyrin IX (PpIX) was separated using the HPLC system (Prominence, Shimadzu, Kyoto, Japan) equipped with a reversed-phase C_18_ column (CAPCELL PAK, C18, SG300, 5 μm, 4.6 mm × 250 mm, Shiseido Co., Ltd., Tokyo, Japan). Elution was started with 10% solvent A and 90% solvent B for 7 min. The elution flow was constant at a rate of 2.0 ml/min. PpIX was continuously detected using a spectrophotometer at 404 nm. The concentrations of the samples were estimated from calibration curves of reference standards.

### Exposure of the cells to light-emitting diode (LED)

Cells (5.0 × 10^3^ cells) were incubated with 1 mM ALA with or without 0.5 mM SFC under 5% CO_2_ at 37°C for 4 h. Cells were then exposed to LED irradiation for 5 min (630 nm, 1080 mJ/cm^2^) by placement of the plate below an LED irradiation unit (provided by SBI Pharma CO., LTD., Tokyo, Japan) as previously described [8]. Cells were further incubated in the dark under 5% CO_2_ at 37°C for 24 h. Cell viability was then measured by the MTT assay as previously described [19].

### Quantitative PCR

NucleoSpin RNA II (MACHEREY-NAGEL, Düren, Mannheim, Germany) was used to extract total RNAs from cells and specimens according to the manufacturer’s protocol. Total RNAs (1 μg) were reverse-transcribed to produce first-strand cDNA using the PrimeScript RT reagent Kit with gDNA Eraser (TaKaRa, Shiga, Japan) according to the manufacturer’s protocol [8,9,18]. The Thermal Cycler Dice Real Time System (TaKaRa, Shiga, Japan) was used for a two-step reverse transcription polymerase chain reaction. The mRNA transcripts were quantified by SYBR Premix ExTaq (TaKaRa, Shiga, Japan).

*mitoferrin 1*-specific primers 5′-TAGCCAACGGGATAGCTGG-3′, 5′-GTGGTGTAGCTCCGGTAGAAG-3′, *mitoferrin 2*-specific primers 5′-CTGCGTGATGTACCCCATCG-3′, 5′-CCTGTTGCTGTGACGTTCAG-3′, and *frataxin*-specific primers 5′-GTGGAGATCTAGGAACCTATG-3′, 5′-TTAAGGCTTTAGTGAGCTCTG-3′ were used. *Ferrochelatase and gapdh* specific primers were purchased from TaKaRa. The amplification conditions included 30 s at 95°C, a run of 45 cycles at 95°C for 5 s, and 60°C for 60 s, followed by dissociation for 15 s at 95°C and 30 s at 60°C and then 15 s at 95°C on a Thermal Cycler Dice Real Time System.

The Thermal Cycler Dice Real Time System analysis software (TaKaRa, Shiga, Japan) was used to analyze the data. The Ct values (cycle threshold) were calculated by the crossing-point method, and the relative quantities of target mRNA expression levels were measured by comparison with a standard curve. The results for each sample were normalized to *gapdh*, a housekeeping gene.

### Measurement of mitochondrial labile iron

The fluorescence indicator of iron ion, rhodamine B-{(1,10-phenanthrolin-5-yl)aminocarbonyl} benzyl ester (RPA) and iron chelator, pyridoxal isonicotinoyl hydrazone (PIH) were used for measure the amount of mitochondrial labile iron ion. The analysis was performed as previously described with some modifications [20,21]. In brief, RPA selectively accumulates in the mitochondria and its fluorescence is quenched by iron. In the presence of an iron chelator, PIH, RPA fluorescence is not quenched. Thus, the amount of mitochondrial labile iron ion is determined by the difference between fluorescence intensities of {RPA} and {RPA+PIH}.

## Results

### The mRNA expression levels of iron-metabolism-related genes in human bladder cancer specimens and tumor cells

**Fig 1:**
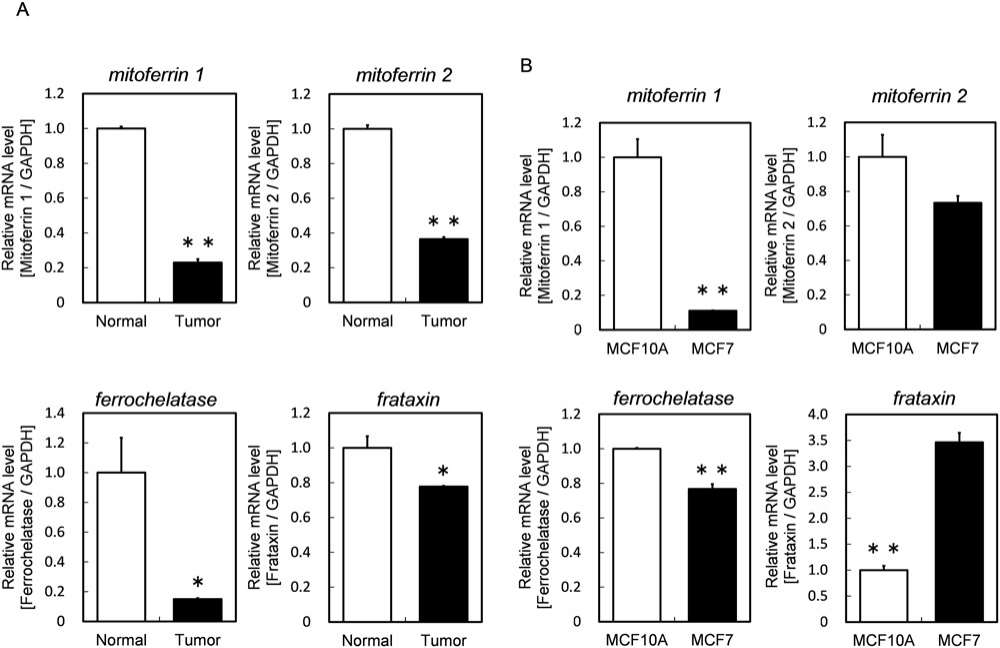
The mRNA expression levels of iron-metabolism-related genes in cancer and normal bladder specimens from same patient (A) and in MCF10A and MCF7 (B). The expression levels of target genes were normalized with *gapdh*, a housekeeping gene. Data are expressed as means ± S.D. in two independent experiments. Statistical significance of difference is indicated by *p <0.05, **p <0.005 determined with Student’s t-test.

The mRNA expression levels of iron-metabolism-related genes in human bladder cancer and normal specimens are summarized in Fig 1A. The expression levels of *mitoferrin 1* and *mitoferrin 2*, which are the mitochondrial iron transporters, were lower in cancer specimens than in normal specimens. The expression levels of *ferrochelatase* and *frataxin*, which transports mitochondrial labile iron ion to *ferrochelatase*, were also lower in cancer specimens. These results suggested that iron metabolism, associated with heme biosynthesis, in tumor cells is lower than that in normal cells.

Furthermore, we investigated the mRNA expressions related to mitochondrial iron metabolism in human breast adenocarcinoma cell line MCF7 as tumor cells and in normal human mammary epithelial cell line MCF10A as normal cells (Fig 1B). As a result, the mRNA expression levels of *mitoferrin 1* and *mitoferrin 2* in MCF7 cells were lower than those in MCF10A cells. In addition, the expression level of *ferrochelarase* was lower in MCF7. These results suggested that the uptake ability of iron ion into mitochondria is lower in MCF7 than in MCF10A.

### Amounts of mitochondrial labile iron ion in cancer cells

**Fig 2:**
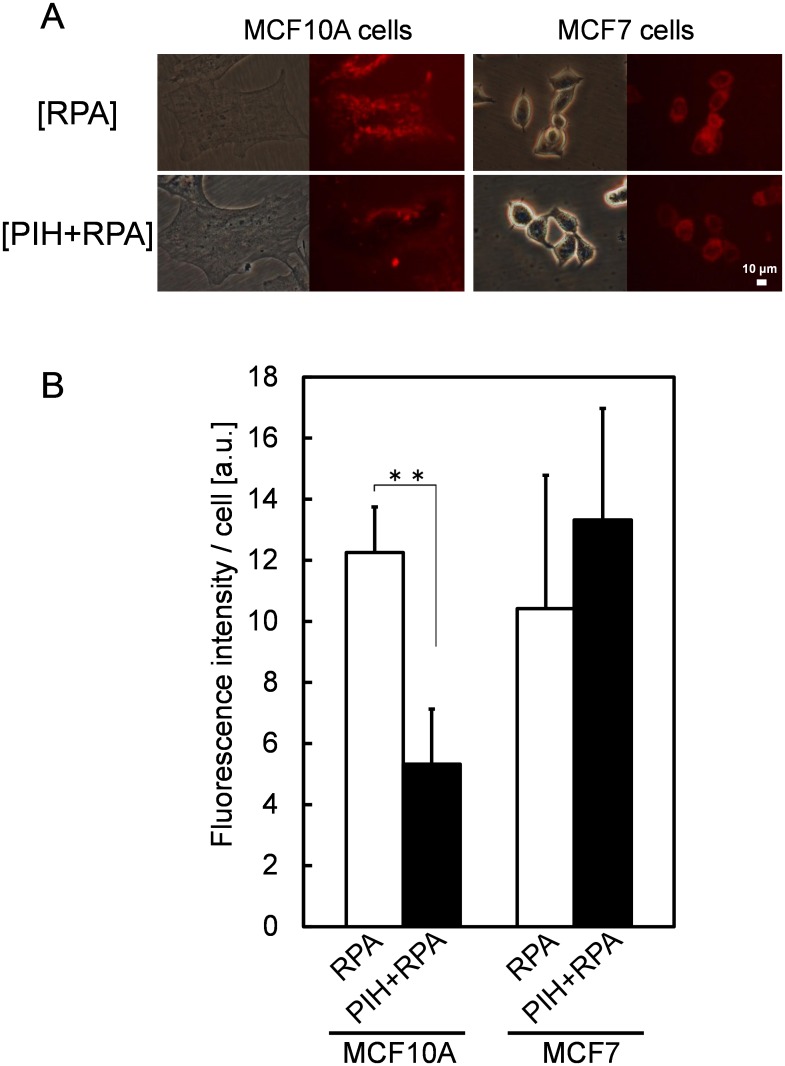
RPA fluorescence images of living MCF10A and MCF7cells (A). The amount of mitochondrial labile iron ion was measured by fluorescence indicator of iron ion, RPA and iron chelator, PIH. The fluorescence intensity of {RPA} indicates the amount of fluorescent probe that did not react with iron ions, whereas {RPA+PIH} indicates the whole amount of incorporated fluorescent probe. Fluorescence intensity were quantified using IMAGE J (B). Data are expressed as means ± S.D. in five independent experiments. Statistical significance of difference is indicated by **p <0.005 determined with Student’s t-test.

To confirm the difference between tumor cells and normal cells in iron ion uptake into mitochondria, we measured the amount of mitochondrial labile iron ion. To measure the iron amount in living cells, we used a fluorescence indicator of iron ion, rhodamine B-{(1,10-phenanthrolin-5-yl)aminocarbonyl} benzyl ester (RPA) and an iron chelator, pyridoxal isonicotinoyl hydrazone (PIH). The fluorescence intensity of {RPA} in Fig 2A and Fig 2B indicates the amount of fluorescent probe that did not react with iron ions, whereas {RPA+PIH} indicates the whole amount of incorporated fluorescent probe. The difference between these fluorescence intensities corresponds to the amount of mitochondrial labile iron ion.

The difference in fluorescence intensities between {RPA} and {RPA+PIH} was less in MCF7 than in MCF10A indicating that the amount of mitochondrial labile iron ion was lower in MCF7 than in MCF10A. This finding agreed with the results of the measurement of mRNA expression levels of *mitoferrins*, which were lower in MCF7 than in MCF10A.

### PpIX accumulation in the presence of excess iron ion

**Fig 3:**
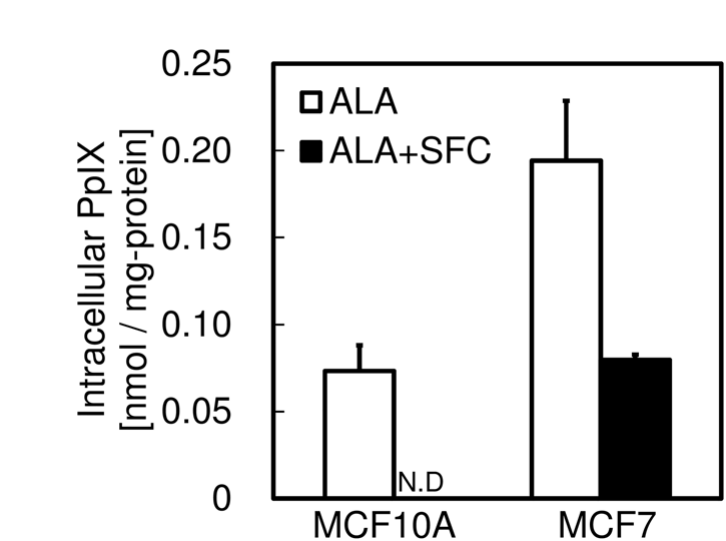
The effect of SFC on intracellular PpIX accumulation levels. Cells were incubated with 1 mM ALA with or without 0.5 mM SFC for 4 h. PpIX accumulation was determined by HPLC analysis as described in EXPERIMENTAL PROCEDURES. Data are expressed as means ± S.D. in three independent experiments.

Fig 3 shows intracellular PpIX accumulations after ALA treatment with or without sodium ferrous citrate (SFC) as a source of iron ion. When only ALA was added to MCF10A cells, PpIX was accumulated to a small extent after 4 h. However, when SFC was added with ALA, PpIX was not detected. In contrast, PpIX was retained in MCF7 even in the presence of SFC. This result indicates that the presence of excess iron ion did not result in the complete metabolism of PpIX to heme in MCF7 cells. This result supports the fingings that the expression levels of *mitoferrins* were lower in MCF7 cells and that the uptake of iron ions into mitochondria was lower in MCF7 than in MCF10A. It also supports the findings that the mRNA expression level of *ferrochelatase* was lower and the ability of PpIX metabolism also lower in MCF7.

### The effect of SFC on the specificity of ALA-PDT

**Fig 4:**
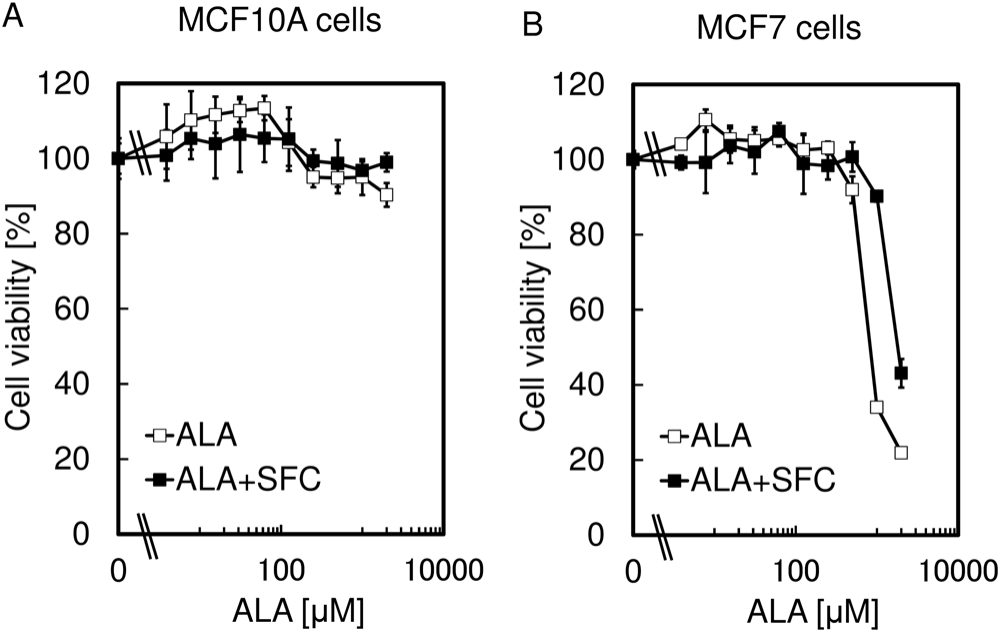
The effect of SFC on the tumor-specificity of ALA-PDT. Cell viability of MCF10A (A) and MCF7 (B) were measured after incubation in the presence of ALA with or without 0.5 mM SFC for 4 h followed by the exposure to LED irradiation for 5 min (630 nm, 1080 mJ / cm^2^.). Data are expressed as means ± S.D. in four independent experiments.

As a next step, cell viability was measured after incubation in the presence of ALA with or without SFC for 4 h followed by the exposure to LED irradiation (Fig 4). As a result, the decrease in cell viability of MCF10A after ALA-PDT was inhibited by the addition of SFC. However, although the decrease of cell viability of MCF7 was also suppressed, it was still very sensitive to ALA-PDT under the presence of SFC. The explanation for this result may be that the addition of SFC eliminated the remaining PpIX by the conversion to heme in MCF10A, whereas PpIX was accumulated in MCF7 (Fig 3). These findings indicated the improvement of the specificity of ALA-PDT effect using SFC.

### The improvement effect of SFC on the specificity of ALA-PDT in various types of cells

**Fig 5:**
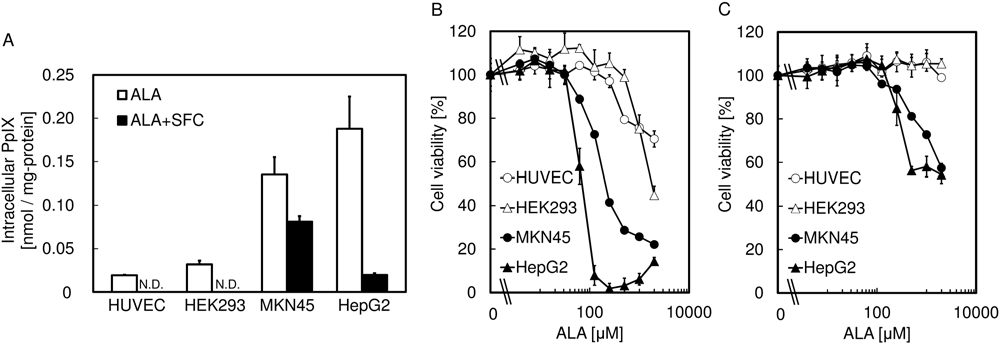
The effect of SFC on the tumor-specificity of ALA-PDT in various types of cells. (A) Intracellular PpIX accumulation levels after 1 mM ALA treatment with or without 0.5 mM SFC. (B) Cell viabilities after ALA-PDT treatment with or without 0.5 mM SFC. Data are expressed as means ± S.D. in four independent experiments.

For further investigation, the effect of SFC on the specificity of ALA-PDT was tested for other cell lines including two human tumor cell lines, MKN45 and HepG2, and two normal cell lines, HUVEC and HEK293 (Fig 5). The results showed that the cell viabilities of the normal cells in the presence of SFC were not reduced, whereas the tumor cell lines showed sensitivity to ALA-PDT even in the presence of SFC. These results suggest that coadministration of SFC with ALA would improve the tumor specificity of the ALA-PDT effect.

## Discussion

The phenomenon of tumor-selective PpIX accumulation after ALA administration is widely used in the clinic. However, the basic understanding of the phenomenon is poor. In previous studies, the activity of ferrochelatase was found to be the key factor in tumor-selective PpIX accumulation [6,7]. In addition, our previous study showed that the amount of PpIX accumulation is dependent on the expressions of transporters involved in the import of ALA and export of PpIX [8,9]. For example, higher expression of dipeptide transporter PEPT1 (SLC15A1), which imports ALA, and lower expression of ATP-binding cassette transporter ABCG2, which exports PpIX, led to higher PpIX accumulation. In addition, SLC6A6 and SLC6A13 mediate the uptake of ALA and may mediate the accumulation of PpIX [10].

Mitochondrial iron metabolism, which is associated with the biosynthesis of heme, is also involved. For instance, overexpression of mitoferrin 2, which transports iron ions into mitochondria, reduced PpIX accumulation [13]. Furthermore, overexpression of frataxin, which transports iron ions to ferrochelatase, reduced cellular PpIX accumulations [14]. Although many factors are involved, the dominant mechanism of tumor-selective PpIX accumulation is unknown.

In the present study, we focused on the mitochondrial labile iron ion, which is the substrate for metabolism of PpIX to heme. First, we investigated the mRNA expression levels of iron-metabolism-related genes in human bladder cancer specimens. As a result, in cancer specimens, the expression levels of the mRNAs were lower than those in normal specimens (Fig 1A). These results suggest that the metabolism of iron is different between cancer and normal cells. Accordingly, we investigated the expression levels of those genes in tumor cells and normal ones, obtaining results similar to those for human specimens (Fig 1B). In addition, the iron levels in mitochondria were lower in tumor cells than those in normal cells (Fig 2). This result agrees with the results of the measurement of mRNA expression levels of *mitoferrins*, which were lower in tumor cells than in normal cells. These results suggest that the amount of mitochondrial labile iron ion plays a key role in tumor-selective PpIX accumulation.

To further investigate this suggestion, we measured the intracellular PpIX accumulation after co-administration of SFC with ALA. We did not observe PpIX accumulation in normal cells, but still observe it in tumor cells (Figs 3 and 5). These results showed that the addition of excess iron ion could not support complete metabolism of PpIX to heme in tumor cells, in agreement with the findings that the expression levels of *mitoferrins* and the uptake abilities of iron ions into mitochondria were lower in tumor than in normal cells. They also supported the findings that the expression level of *ferrochelatase* and the abilities of PpIX metabolism were both lower in tumor cells.

Cell viabilities were measured after incubation in the presence of ALA with SFC for 4 h, followed by exposure to LED irradiation. As a result, decreases in cell viability of normal cells after ALA-PDT were not observed with the addition of SFC. In contrast, although the decrease of cell viability of tumor cells was also suppressed, it was still very sensitive to ALA-PDT in the presence of SFC (Figs 4 and 5). The explanation of this result could be that the addition of SFC eliminated the remaining PpIX by the conversion to heme in normal cells, whereas PpIX was accumulated in tumor cells (Figs 3 and 5). These findings give a clue for the improvement of the specificity of ALA-PDT effect using SFC.

In the previous studies, iron chelating agents were used to make ALA-PDT more efficient. For instance, PpIX accumulation was increased and ALA-PDT effect was improved by addition of the iron chelators deferoxamine or EDTA [22,23]. Based on these studies, it was considered that chelating iron ion was crucial for ALA-PDT. Moreover, Polanski et al reported iron complexing agent thiosemicarbazones showed high synergistic effect of ALA-PDT [24,25]. However, in the present study, we have proposed a novel strategy that improves the specificity of ALA-PDT by adding iron. In future studies, we propose to identify the optimal concentration of iron to improve both the specificity and efficiency of ALA-PDT. In addition, we expect that compounds associated with iron metabolism may be used as well as iron-chelating agents to improve ALA-PDT.
